# Impact of Alcohol Consumption on Multiple Sclerosis Using Model-based Standardization and Misclassification Adjustment Via Probabilistic Bias Analysis

**DOI:** 10.34172/aim.2023.83

**Published:** 2023-10-01

**Authors:** Pooneh Malekifar, Saharnaz Nedjat, Ibrahim Abdollahpour, Maryam Nazemipour, Saeed Malekifar, Mohammad Ali Mansournia

**Affiliations:** ^1^Department of Epidemiology and Biostatistics, School of Public Health, Tehran University of Medical Sciences, Tehran, Iran; ^2^Child Growth and Development Research Center, Research Institute for Primordial Prevention of Non-Communicable Disease, Isfahan University of Medical Science, Isfahan, Iran; ^3^Department of Computer Engineering, Iran University of Science and Technology, Tehran, Iran

**Keywords:** Alcohol consumption, G-formula, Model-based standardization, Monte Carlo sensitivity analysis, Multiple sclerosis, Probabilistic bias analysis

## Abstract

**Background:**

The etiology of multiple sclerosis (MS) is still not well-demonstrated, and assessment of some risk factors like alcohol consumption has problems like confounding and measurement bias. To determine the causal effect of alcohol consumption on MS after adjusting for alcohol consumption misclassification bias and confounders.

**Methods:**

In a population-based incident case-control study, 547 patients with MS and 1057 healthy people were recruited. A minimally sufficient adjustment set of confounders was derived using the causal directed acyclic graph. The probabilistic bias analysis method (PBAM) using beta, logit-logistic, and triangular probability distributions for sensitivity/specificity to adjust for misclassification bias in self-reporting alcohol consumption and model-based standardization (MBS) to estimate the causal effect of alcohol consumption were used. Population attributable fraction (PAF) estimates with 95% Monte Carlo sensitivity analysis (MCSA) intervals were calculated using PBAM and MBS analysis. Bootstrap was used to deal with random errors.

**Results:**

The adjusted risk ratio (95% MCSA interval) from the probabilistic bias analysis and MBS between alcohol consumption and MS using the three distribution was in the range of 1.93 (1.07 to 4.07) to 2.02 (1.15 to 4.69). The risk difference (RD) in all three scenarios was 0.0001 (0.0000 to 0.0005) and PAF was in the range of 0.15 (0.010 to 0.50) to 0.17 (0.001 to 0.47).

**Conclusion:**

After adjusting for measurement bias, confounding, and random error alcohol consumption had a positive causal effect on the incidence of MS.

## Introduction

 Multiple sclerosis (MS) is the most common non-traumatic disabling disease in young adults.^1,2^ Life expectancy in these patients is 10 years less than the normal population.^3^ Despite its low prevalence, a similar increasing trend has been reported in almost all parts of the world. From 1990 to 2016, the age-standardized prevalence of the disease increased by 10.4%.^4^ Iran has a medium-to-high prevalence of MS; nevertheless, a dramatic increase has been recently reported in its incidence and prevalence.^5-7^

 Numerous studies have been performed to determine the risk factors of MS, but its etiology is still not well-demonstrated. Vitamin D deficiency,^8^ exposure to ultraviolet B (UVB) light,^9^ Epstein-Barr virus (EBV) infection,^10^ smoking,^11^ waterpipe smoking,^12^ diet^13^ and drug abuse^14^ are all associated with the disease. Another suggested risk factor is alcohol consumption. However, there is no conclusive evidence about the possible association between alcohol intake and MS.^15,16^

 The underlying social stigma in societies like Iran may result in the possibility of misclassification or underreporting bias when investigating the consumption of illegal substances including alcohol intake or drug abuse.^17^ Thus, appropriate control for this type of misclassification bias is prudent for a correct causal analysis of alcohol intake and MS diagnosis. The probabilistic bias analysis method (PBAM) is one of the recently emerging methods which provides bias-corrected effect estimates using prior distribution for sensitivity and specificity of exposure misclassification.^18-20^ Bayesian methods (BM) and PBAM often provide similar results though the latter is more straightforward and accessible than BM.^21,22^

 There are several causal methods including inverse probability-of-treatment weighting (IPTW), and model-based standardization (MBS), known as parametric g-formula in the time-varying setting, for estimating the marginal causal effects in cohort and case-control studies.^14,23-29^ These methods might be appropriate for estimating the causal effect of alcohol consumption on MS, as policy interventions for reducing excessive alcohol consumption at the population level could be more effective.^30^ Therefore, by conducting a large population-based incident case-control study with known case and control sampling fractions, we aimed to evaluate the causal effect of alcohol consumption on MS after adjusting for misclassification bias and confounders using PBAM and MBS.

## Materials and Methods

###  Design and Sampling

 This case-control study was conducted in Tehran, Iran.^12,31^ All participants provided verbal informed consent, and all stages of this study were based on the Helsinki Declaration. The study base was individuals aged 15–50 years who were residents in Tehran between August 2013 and February 2015. The case group consisted of 547 new cases with MS, definitively diagnosed by at least one neurologist using the 2010 McDonald criteria as well as MRI confirmation. A random-digit dialing (RDD) sampling technique was used for control selection, and 1057 alive person aged 15–50 years were selected. The international physical activity and a validated Persian version of EnvIMS-Q questionnaires along with the neurologist’s opinion were used to prepare the MS comprehensive checklist.^32-35^ Lifestyle data and other confounding variables were collected via interviews. Ten interviewers were identified based on their skill set and trained to use the standardized data collection procedures. Phone interviews were conducted and we monitored the data collection activities for any interviewer bias by randomly recording interviews. To decrease the possibility of misclassification of undetected cases, the clinical signs and characteristics of MS were explained to the control sample. Data gathering in case and control groups was accomplished with the same protocol. Drinking any type of alcohol (beer, wine, liquor, and the other types) for at least 6 times in at least a six month period was considered as the life time alcohol consumption. Life events were defined as significant stressful occurrences in the past 4-5 years, including but not limited to divorce, migration, and loss of loved ones.

###  Statistical Analysis

**Figure 1:**
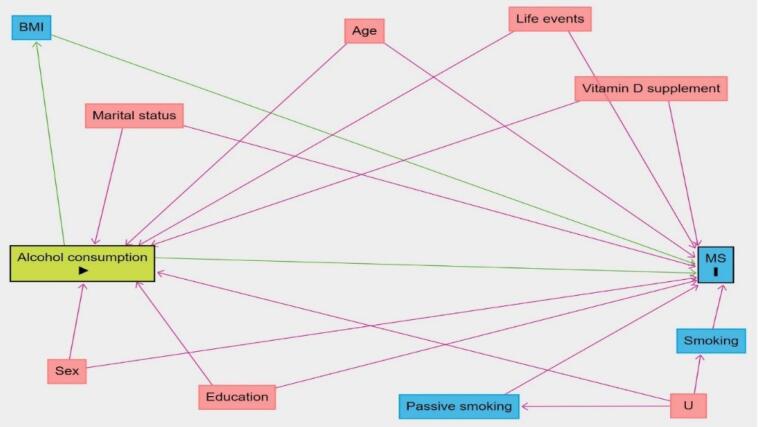
Causal Directed Acyclic Graph for the Effect of Alcohol Consumption on Multiple Sclerosis. The minimally sufficient adjustment set of confounders consists of age, sex, marital status, education, smoking, passive smoking, life events and vitamin D supplement

 A literature review was performed to determine the potential confounders of the causal relationship between alcohol consumption and MS. The causal directed acyclic graph^36-44^ for the effect of alcohol consumption on MS is presented in Figure 1. We used Pearl’s back-door criterion to identify a minimally sufficient set of confounders for adjustment. All analyses were performed using the R statistical software.

###  Bias and Causal Analysis Using PBAM and MBS

 Step 1: To parameterize probability distributions for sensitivity/specificity, the literature was systematically reviewed in Scopus, PubMed, and Web of Science using the keywords “accuracy”, “measurement error”, “measurement bias”, “sensitivity”, “specificity”, “validity”, “self-reported alcohol”, and “multiple sclerosis”. Sensitivity and specificity estimates along with their 95% confidence intervals (CIs) were extracted from the studies. Pooled sensitivity and specificity values were calculated using the random-effects model.^45^

 Step 2: Six studies with eight sensitivity and specificity values were derived based on the literature review.^46-51^ Pooled sensitivity and specificity estimates with 95% CIs were 0.79 (0.72, 0.86) and 0.84 (0.80, 0.89), respectively. These studies included patients who visited the emergency department, individuals with addiction, patients infected with HIV, pregnant women, students, and the general population. Since none of the studies included patients with MS, we were unable to estimate differential misclassification, and also none of these studies were conducted in Iran. Objective biomarkers, such as hair analysis tests and various blood tests, served as the gold standard for determining alcohol consumption.

 Step 3: Three probability distributions, namely beta, logit-logistic, and triangular, were specified to represent a diverse range of choices in the absence of empirical information (Supplementary file 1, Figure S1). They were specified so that their median (2.5th and 97.5th percentiles) was equal to the pooled estimate (95% CI) obtained in the previous step. Parameters for triangular, beta, and logit-logistic distributions are shown in Table 1.

**Table 1:** Probability Distributions Parameters for Triangular, Beta and Logistic Distributions in Case and Control Groups

**Bias Parameters**	**Triangular Distribution** **(Minimum; Maximum; Mode)**	**Beta Distribution** **(Alpha; Beta)**	**Logit-Logistic Distribution** (Location; Scale)^*^
Sensitivity	0.72, 0.86, 0.79	79.79, 20.32	0.79, 0.004
Specificity	0.80, 0.89, 0.84	88.75, 16.03	0.84, 0.003

^*^The location and scale for logistic distribution are presented, and the logit-logistic distribution is the expit transformation of this distribution.

 Step 4: The sensitivity and specificity matrix as well as the numbers of reported exposed and unexposed cases were used to obtain the expected and unexposed true number of cases (for more explanations, see Figure S1). Sensitivity and specificity values were randomly selected from the probability distributions discussed in the third step.

 Step 5: Positive predictive value (PPV) and negative predictive value (NPV) were obtained (for more explanations, see Figure S1). Negative or zero A or B values were discarded and the fourth and fifth steps were repeated.

 Step 6: The expected exposure status for each person was obtained using the observed exposure status, PPV, and NPV values. This variable was assumed to be Bernoulli-distributed with the probability parameters equal to PPV and NPV for exposed and unexposed cases. So, we generated a random variable with a uniform distribution (Ui) between the values of zero and one. For the exposed case (reported alcohol), the exposure status was set to be 0 if Ui > PPV; otherwise, the exposure status was not changed. For the unexposed cases, the exposure status was set to be 1 if Ui > NPV; otherwise, the exposure status was not changed.

 Step 7: A multivariable logistic regression model was fitted with case/control status as the response variable, and the imputed exposure status derived in the previous step, as well as confounders as predictors. Fractional polynomials were used to assess the linearity assumption for the variable age.

 Step 8: Using the logistic regression model from the previous step, the MBS was performed by calculating the standardized risks in the exposed and unexposed over the confounders’ distributions in the study base of the case and control groups, obtained by model-intercept correction. First, the bias in the intercept due to case-control sampling was corrected by subtracting the log (sampling fraction in case/sampling fraction in control) i.e. log ( $\frac{0.96}{1057/5115679}$ ) = 8.44, from the apparent intercept. The sample fraction in the case group refers to the ratio of the number of cases to the total number of individuals with MS, and the sample fraction in the control group represents the ratio of the number of controls to the number of general population aged between 15 and 50 years. Second, the standardized risk in the exposed was calculated by predicting the probability of outcome based on the observed confounder values and setting all participants to be exposed and then averaging the predicted risks, weighted by inverse sampling fractions in the case and control groups. The standardized risk in the unexposed was calculated similarly by setting all unexposed. Marginal risk ratio (RR) and risk difference (RD) were obtained by dividing and subtracting the calculated standardized risks in the exposed and unexposed.

 Step 9: The results of the previous step constitute one round. Steps 4–8 were repeated 1000 times to obtain a simulation interval using probabilistic bias analysis via Monte Carlo simulation from the distributions mentioned in step 3: the median (50th percentile), and 2.5 and 97.5 percentiles of the RRs and RDs obtained were considered as the point estimate and Monte Carlo sensitivity analysis (MCSA) limits, respectively.

 By now, we corrected misclassification bias and confounding, but random error should be addressed as well. Therefore, all steps 4 to 9 were performed in each of the 500 bootstrap sample, reconstructed through separately sampling by replacement from cases and controls with the same original sizes, yielding 500 000 RRs and RDs corrected for both misclassification/confounding and random error.

###  Population Attributable Fraction 

 To determine the fraction of all MS patients in the population that might be attributable to alcohol consumption, we calculated population attributable fraction (PAF) as follows^52-54^:


$$PAF=\frac{\mathrm{Pr}\left[Y=1\right]-\mathrm{Pr}\left[Y^{a=0}=1\right]}{\mathrm{Pr}\left[Y=1\right]}$$

where Pr [Y = 1] is the observed risk and Pr [Y^a=0^ = 1] refers to the risk that would have been observed if everybody had received a = 0 or no alcohol consumption. Pr [Y^a=0^ = 1] was calculated as the standardized risk in the unexposed, explained in step 8. For conventional analysis, we used the PAF formula above, provided that there was no adjustment for misclassification in calculating observed risk and risk under no alcohol consumption assumption i.e. the analysis starts from step 7 above using the observed exposure (instead of the imputed exposure) and continues through step 8 in which no Monte Carlo simulation was performed, and CI was obtained using 500 bootstrap samples.

## Results

 This case-control study included 547 cases and 1057 controls, of whom 401 (73.3%) and 544 (51.5%) were female, respectively. The mean age (SD) was 30.5 (7.53) for cases and 31.3 (9.33) for controls. The characteristics of both groups are presented in Table 2. According to Figure 1, the variables age, sex, marital status, education, smoking, passive smoking, life events, and vitamin D supplement were considered to be confounders for the effect of alcohol consumption on MS.

**Table 2:** Characteristics of Cases and Controls

**Variables**	**MS cases**	**Controls**
Age (years); mean (SD)	30.5 (7.5)	31.3 (9.3)
Gender (female)^*^	401 (73.3)	544 (51.5)
Marital status		
Single	246 (45.1)	486 (46.2)
Married	300 (54.9)	567 (53.8)
Educational year; mean (SD)	13.8 (3.3)	13.4 (3.2)
Smoking (yes)	108 (19.7)	211 (19.9)
Passive smoking (yes)	285 (52.1)	396 (37.5)
Life event (yes)	442 (80.8)	866 (81.9)
Vitamin D supplement (yes)	14 (0.02)	80 (0.07)

MS, multiple sclerosis; SD, standard deviation. ^*^No. (%) was reported unless otherwise stated.

**Figure 2:**
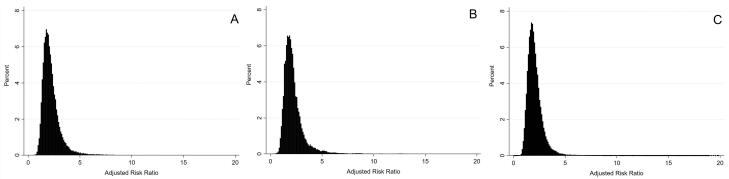
Triangular (A), Beta (B) or Logit-Logistic (C) Distributions of RRs, Adjusted for Measurement Bias, Confounding and Random Error

 The confounder-adjusted odds ratio (OR) obtained from the conventional logistic regression analysis for the effect of alcohol consumption on MS was 1.75 (95% CI: 1.28 to 2.30). Table 3 presents the results of the PBAM-and-MBS analysis. After adjustment for confounding and misclassification bias, the RR estimates (95% MCSA interval) were 2.02 (1.15 to 4.69), 1.99 (1.06 to 5.54), and 1.93 (1.07 to 4.07), considering triangular, beta and logit-logistic distributions for bias parameters, respectively. The distribution of misclassification bias- and confounders-adjusted RRs using different bias parameters is demonstrated in Figure 2.

**Table 3:** Adjusted Risk Ratio and Risk Difference Estimates with 95% MCSA Interval Using Probabilistic Bias and MBS Analyses

**Bias Parameter Distribution**	**Effect Measure Estimate (95% MCSA)**	
	**RR**	**RD**
Triangular	2.02 (1.15 to 4.69)	0.0001 (0.0000 to 0.0005)
Beta	1.99 (1.06 to 5.54)	0.0001 (0.0000 to 0.0005)
Logit-logistic	1.93 (1.07 to 4.07)	0.0001 (0.0000 to 0.0005)

CI, confidence interval; MCSA, Monte Carlo sensitivity analysis; RR, risk ratio; RD, risk difference.

 The PAF (95% CI) for alcohol assumption was 0.12 (0.05 to 0.18) using conventional analysis. Table 4 shows PAF estimates with a 95% MCSA interval using PBAM and MBS analysis. PAF (95% MCSA interval) was 0.17 (0.001 to 0.47) using triangular parameter distribution, 0.16 (0.014 to 0.47) using beta and 0.15 (0.010 to 0.50) logit-logistic bias parameter distribution.

**Table 4:** Estimates of Population Attributable Fraction with 95% MCSA Intervals Using Probabilistic Bias and MBS Analyses

**Bias Parameter Distribution**	**Bias Analysis (95% MCSA)**
Triangular	0.17 (0.001 to 0.47)
Beta	0.16 (0.014 to 0.47)
Logit-logistic	0.15 (0.010 to 0.50)

## Discussion

 By conducting a large population-based incident case-control study, we assessed the causal effect of alcohol consumption on MS after adjusting for three main sources of error i.e. misclassification, confounding, and random error. Based on our study, after adjusting for errors, alcohol consumption has a causal effect on MS. The PBAM was used to address misclassification in the self-reported alcohol consumption.^21^ This method depends on the sensitivity and specificity estimates obtained from resources such as a literature review and meta-analysis.^21,55^ Three different distributions were used to assess the sensitivity of the results to such choices.

 PBAM has been used to correct misclassification in some epidemiological studies. Livingston et al compared three methods of regression calibration, multiple imputations for measurement error, and PBAM to correct measurement errors in self-reported adolescent alcohol use.^56^ The results of this study indicate that PBAM is not a good method for the correction when the sample size is small. Due to the sparseness of the crosstab cells used to estimate the sensitivity and specificity, the performance of the PBAM varied greatly with the sample size. Also, PBAM had great performance when sensitivity and specificity were high. Pakzad et al assessed the association between smoking and breast cancer adjusted for confounders and self-reported smoking misclassification using PBAM.^20^ In that study, OR estimate increased from 0.64 for conventional analysis to ranges of 2.63–2.69 and 1.73–2.83 for non-differential and differential misclassification in PBAM. So, non-significant negative adjusted association between smoking and breast cancer changed to a significant positive adjusted association. In a recent study by Pakzad et al, adjustment for misclassification in the self-reported alcohol consumption using PBAM changed no evidence against independence between alcohol consumption and breast cancer to a substantial positive association.^57^ Bodnar et al applied PBAM to correct misclassification in self-reported pre-pregnancy BMI category in the assessment of the association between BMI and pregnancy outcomes, and showed that misclassification adjustment attenuates the unadjusted association.^58^

 To our knowledge, this study is the first which combines PBAM with MBS to estimate the causal effect of alcohol consumption on MS. Observational studies are prone to confounding bias, and association does not ensure causation.^59^ IPTW, MBS, and targeted maximum likelihood estimation (TMLE) are the methods that can be used in the case-control studies to estimate the so-called marginal (population-averaged) causal effects.^60^ MBS is a generalization of classical standardization which uses standard outcome regression modeling and standardization.^61,62^

 Parametric g-formula has been used in some epidemiological studies for calculating the causal effects. Taubman et al performed the first large-scale application of the parametric g-formula in 2009, using data from the Nurses’ Health Study to estimate lifestyle interventions on the risk of coronary heart disease, and facilitated future use by making the software available.^63^ Some other researchers like Jain et al,^64^ Westreich et al,^65^ Garcia-Aymerich et al,^66^ Edwards et al,^67^ Murray et al,^68^ and Young et al,^69^ used g-formula and described its pros and cons. In several studies, Mansournia and colleagues applied MBS in case-control studies and parametric g-formula in cohort studies in the fixed and time-varying settings.^23,70-74^

 In this study, we used MBS after probabilistic bias correction of the exposure and obtained RR estimates in range of 1.93–2.02 based on three bias parameter distributions. In fact, alcohol consumption misclassification led to the underestimation in the association of alcohol consumption and MS, and adjusted estimates were increased after correction (OR estimate was 1.75 in conventional analysis). Based on our study results, there is sufficient evidence against the independency of alcohol consumption and MS, and alcohol consumption had a causal effect on MS incidence. A few studies have examined the effect of alcohol consumption on the risk of MS, and reported inconsistent results. A case-control study in Serbia reported a significant association between the consumption of hard liquor per day and the risk of MS (OR = 6.7).^75^ Foster et al showed a relationship between the duration of alcohol consumption and disability and MRI measures in MS. The study included two separate cohorts from Nurses’ Health Study and Nurses’ Health Study II and reported no association between alcohol consumption and MS risk.^76^ On the other hand, Hedström et al merged two case-control studies (EIMS and GEMS study) and concluded that there is an inverse dose-dependent association between alcohol consumption with MS. Women and men who reported high alcohol consumption had lower risk of developing MS compared with nondrinking women and men.^15^

 In this study, we calculated PAF for alcohol consumption based on its definition, i.e. (O - E)/O where O is the observed number of cases and E is the expected number under no exposure in the population. Measurement error in O was adjusted with PBAM and E was calculated using MBS analysis; in our study, measurement error is adjusted with PBAM, and the risk ratio is calculated from the MBS analysis. Based on our result, the PAF estimate for alcohol consumption was in the range of 0.15 to 0.17. No other study has calculated the PAF for alcohol consumption in MS.

 The strengths of this study included a systematic search for bias parameters and using different distributions for them, using fractional polynomials to adjust for age, identification of confounders using a causal diagram, correction of the intercept for using the MBS in the case-control study, and finally, unifying the PBAM and MBS for the causal analysis of case-control studies for the first time.

 The study suffers from some limitations. First, because of the absence of a study reporting alcohol consumption sensitivity and specificity in people with MS, we performed bias correction only under the non-differential scenario; a differential scenario may have had different results. However, the impact of cultural and religious norms and values on alcohol drinking is expected to be independent of MS status and therefore non-differential. Second, alcohol consumption was considered dichotomous which could reduce the study’s statistical power and result in a biased impression of dose-response.^26^ Another limitation is the possibility of unmeasured confounders, as well as measurement error in the confounders such as smoking which may result in residual confounding, and the direction of bias cannot be predicted due to the correlation between measurement errors in alcohol consumption and smoking. This confounding could be assessed in future studies.

## Conclusion

 Bias and causal analysis in this study showed that alcohol consumption doubles the risk of MS, and a significant proportion of MS cases in Iran are linked to alcohol consumption.

## Supplementary Files

Supplementary file 1 contains Figure S1.

## References

[1] Kobelt G, Thompson A, Berg J, Gannedahl M, Eriksson J (2017). New insights into the burden and costs of multiple sclerosis in Europe. Mult Scler.

[2] Reich DS, Lucchinetti CF, Calabresi PA (2018). Multiple sclerosis. N Engl J Med.

[3] Thormann A, Sørensen PS, Koch-Henriksen N, Laursen B, Magyari M (2017). Comorbidity in multiple sclerosis is associated with diagnostic delays and increased mortality. Neurology.

[4] Wallin MT, Culpepper WJ, Nichols E, Bhutta ZA, Gebrehiwot TT, Hay SI (2019). Global, regional, and national burden of multiple sclerosis 1990-2016: a systematic analysis for the Global Burden of Disease Study 2016. Lancet Neurol.

[5] Etemadifar M, Izadi S, Nikseresht A, Sharifian M, Sahraian MA, Nasr Z (2014). Estimated prevalence and incidence of multiple sclerosis in Iran. Eur Neurol.

[6] Eskandarieh S, Heydarpour P, Elhami SR, Sahraian MA (2017). Prevalence and incidence of multiple sclerosis in Tehran, Iran. Iran J Public Health.

[7] Etemadifar M, Maghzi AH (2011). Sharp increase in the incidence and prevalence of multiple sclerosis in Isfahan, Iran. Mult Scler.

[8] Duan S, Lv Z, Fan X, Wang L, Han F, Wang H (2014). Vitamin D status and the risk of multiple sclerosis: a systematic review and meta-analysis. Neurosci Lett.

[9] Vitkova M, Diouf I, Malpas C, Horakova D, Kubala Havrdova E, Patti F (2022). Association of latitude and exposure to ultraviolet B radiation with severity of multiple sclerosis: an international registry study. Neurology.

[10] Jacobs BM, Giovannoni G, Cuzick J, Dobson R (2020). Systematic review and meta-analysis of the association between Epstein-Barr virus, multiple sclerosis and other risk factors. Mult Scler.

[11] Degelman ML, Herman KM (2017). Smoking and multiple sclerosis: a systematic review and meta-analysis using the Bradford Hill criteria for causation. Mult Scler Relat Disord.

[12] Abdollahpour I, Nedjat S, Sahraian MA, Mansournia MA, Otahal P, van der Mei I (2017). Waterpipe smoking associated with multiple sclerosis: a population-based incident case-control study. Mult Scler.

[13] Abdollahpour I, Jakimovski D, Shivappa N, Hébert JR, Vahid F, Nedjat S (2020). Dietary inflammatory index and risk of multiple sclerosis: findings from a large population-based incident case-control study. Clin Nutr.

[14] Abdollahpour I, Nedjat S, Mansournia MA, Schuster T (2018). Estimation of the marginal effect of regular drug use on multiple sclerosis in the Iranian population. PLoS One.

[15] Hedström AK, Hillert J, Olsson T, Alfredsson L (2014). Alcohol as a modifiable lifestyle factor affecting multiple sclerosis risk. JAMA Neurol.

[16] D’Hooghe M B, Haentjens P, Nagels G, De Keyser J (2012). Alcohol, coffee, fish, smoking and disease progression in multiple sclerosis. Eur J Neurol.

[17] Carlsson S, Hammar N, Hakala P, Kaprio J, Marniemi J, Rönnemaa T (2003). Assessment of alcohol consumption by mailed questionnaire in epidemiological studies: evaluation of misclassification using a dietary history interview and biochemical markers. Eur J Epidemiol.

[18] Fox MP, Lash TL, Greenland S (2005). A method to automate probabilistic sensitivity analyses of misclassified binary variables. Int J Epidemiol.

[19] Lash TL, Fox MP, Thwin SS, Geiger AM, Buist DS, Wei F (2007). Using probabilistic corrections to account for abstractor agreement in medical record reviews. Am J Epidemiol.

[20] Pakzad R, Nedjat S, Yaseri M, Salehiniya H, Mansournia N, Nazemipour M (2020). Effect of smoking on breast cancer by adjusting for smoking misclassification bias and confounders using a probabilistic bias analysis method. Clin Epidemiol.

[21] MacLehose RF, Gustafson P (2012). Is probabilistic bias analysis approximately Bayesian?. Epidemiology.

[22] Howe CJ, Cole SR (2009). Applying quantitative bias analysis to epidemiologic Data: By Timothy L Lash, Matthew P Fox, and Aliza K Fink. Am J Epidemiol.

[23] Mansournia MA, Etminan M, Danaei G, Kaufman JS, Collins G (2017). Handling time varying confounding in observational research. BMJ.

[24] Mansournia MA, Altman DG (2016). Inverse probability weighting. BMJ.

[25] Mansournia MA, Naimi AI, Greenland S (2019). The implications of using lagged and baseline exposure terms in longitudinal causal and regression models. Am J Epidemiol.

[26] Mansournia MA, Danaei G, Forouzanfar MH, Mahmoodi M, Jamali M, Mansournia N (2012). Effect of physical activity on functional performance and knee pain in patients with osteoarthritis: analysis with marginal structural models. Epidemiology.

[27] Koohi F, Khalili D, Soori H, Nazemipour M, Mansournia MA (2022). Longitudinal effects of lipid indices on incident cardiovascular diseases adjusting for time-varying confounding using marginal structural models: 25 years follow-up of two US cohort studies. Glob Epidemiol.

[28] Abdollahpour I, Nedjat S, Almasi-Hashiani A, Nazemipour M, Mansournia MA, Luque-Fernandez MA (2021). Estimating the marginal causal effect and potential impact of waterpipe smoking on risk of multiple sclerosis using the targeted maximum likelihood estimation method: a large, population-based incident case-control study. Am J Epidemiol.

[29] Almasi-Hashiani A, Nedjat S, Mansournia MA (2018). Causal methods for observational research: a primer. Arch Iran Med.

[30] Elder RW, Lawrence B, Ferguson A, Naimi TS, Brewer RD, Chattopadhyay SK (2010). The effectiveness of tax policy interventions for reducing excessive alcohol consumption and related harms. Am J Prev Med.

[31] Abdollahpour I, Nedjat S, Mansournia MA, Sahraian MA, van der Mei I (2018). Lifestyle factors and multiple sclerosis: a population-based incident case-control study. Mult Scler Relat Disord.

[32] Pugliatti M, Harbo HF, Holmøy T, Kampman MT, Myhr KM, Riise T (2008). Environmental risk factors in multiple sclerosis. Acta Neurol Scand Suppl.

[33] Craig CL, Marshall AL, Sjöström M, Bauman AE, Booth ML, Ainsworth BE (2003). International physical activity questionnaire: 12-country reliability and validity. Med Sci Sports Exerc.

[34] Pugliatti M, Casetta I, Drulovic J, Granieri E, Holmøy T, Kampman MT, et al. A questionnaire for multinational case-control studies of environmental risk factors in multiple sclerosis (EnvIMS-Q). Acta Neurol Scand Suppl. 2012(195):43-50. 10.1111/ane.12032. 23278656

[35] Sahraian MA, Naghshineh H, Shati M, Razeghi Jahromi S, Rezaei N (2016). Persian adaptation of a questionnaire of environmental risk factors in multiple sclerosis (EnvIMS-Q). Mult Scler Relat Disord.

[36] Mansournia MA, Higgins JP, Sterne JA, Hernán MA (2017). Biases in randomized trials: a conversation between trialists and epidemiologists. Epidemiology.

[37] Mansournia MA, Hernán MA, Greenland S (2013). Matched designs and causal diagrams. Int J Epidemiol.

[38] Etminan M, Collins GS, Mansournia MA (2020). Using causal diagrams to improve the design and interpretation of medical research. Chest.

[39] Kyriacou DN, Greenland P, Mansournia MA (2023). Using causal diagrams for biomedical research. Ann Emerg Med.

[40] Etminan M, Brophy JM, Collins G, Nazemipour M, Mansournia MA (2021). To adjust or not to adjust: the role of different covariates in cardiovascular observational studies. Am Heart J.

[41] Mansournia MA, Nazemipour M, Etminan M (2021). Causal diagrams for immortal time bias. Int J Epidemiol.

[42] Mansournia MA, Nazemipour M, Etminan M (2022). Interaction contrasts and collider bias. Am J Epidemiol.

[43] Etminan M, Nazemipour M, Candidate MS, Mansournia MA (2021). Potential biases in studies of acid-suppressing drugs and COVID-19 infection. Gastroenterology.

[44] Mansournia MA, Nazemipour M, Etminan M (2022). Time-fixed vs time-varying causal diagrams for immortal time bias. Int J Epidemiol.

[45] Harris RJ, Deeks JJ, Altman DG, Bradburn MJ, Harbord RM, Sterne JA (2008). Metan: fixed-and random-effects meta-analysis. Stata J.

[46] Woo SH, Lee WJ, Jeong WJ, Kyong YY, Choi SM (2013). Blood alcohol concentration and self-reported alcohol ingestion in acute poisoned patients who visited an emergency department. Scand J Trauma Resusc Emerg Med.

[47] Whitford JL, Widner SC, Mellick D, Elkins RL (2009). Self-report of drinking compared to objective markers of alcohol consumption. Am J Drug Alcohol Abuse.

[48] Iglesias K, Lannoy S, Sporkert F, Daeppen JB, Gmel G, Baggio S (2020). Performance of self-reported measures of alcohol use and of harmful drinking patterns against ethyl glucuronide hair testing among young Swiss men. PLoS One.

[49] Bajunirwe F, Haberer JE, Boum Y 2nd, Hunt P, Mocello R, Martin JN (2014). Comparison of self-reported alcohol consumption to phosphatidylethanol measurement among HIV-infected patients initiating antiretroviral treatment in southwestern Uganda. PLoS One.

[50] Bertol E, Vaiano F, Boscolo-Berto R, Fioravanti A, Palumbo D, Catalani V (2017). Alcohol, caffeine, and nicotine consumption in adolescents: hair analysis versus self-report. Am J Drug Alcohol Abuse.

[51] Himes SK, Dukes KA, Tripp T, Petersen JM, Raffo C, Burd L (2015). Clinical sensitivity and specificity of meconium fatty acid ethyl ester, ethyl glucuronide, and ethyl sulfate for detecting maternal drinking during pregnancy. Clin Chem.

[52] Khosravi A, Nielsen RO, Mansournia MA (2020). Methods matter: population attributable fraction (PAF) in sport and exercise medicine. Br J Sports Med.

[53] Khosravi A, Nazemipour M, Shinozaki T, Mansournia MA (2021). Population attributable fraction in textbooks: Time to revise. Glob Epidemiol.

[54] Mansournia MA, Altman DG (2018). Population attributable fraction. BMJ.

[55] Lash TL, Fink AK (2003). Semi-automated sensitivity analysis to assess systematic errors in observational data. Epidemiology.

[56] Livingston MD III, Cannell B, Muller K, Komro KA (2018). Comparing methods of misclassification correction for studies of adolescent alcohol use. Am J Drug Alcohol Abuse.

[57] Pakzad R, Nedjat S, Salehiniya H, Mansournia N, Etminan M, Nazemipour M (2023). Effect of alcohol consumption on breast cancer: probabilistic bias analysis for adjustment of exposure misclassification bias and confounders. BMC Med Res Methodol.

[58] Bodnar LM, Siega-Riz AM, Simhan HN, Diesel JC, Abrams B (2010). The impact of exposure misclassification on associations between prepregnancy BMI and adverse pregnancy outcomes. Obesity (Silver Spring).

[59] Nørgaard M, Ehrenstein V, Vandenbroucke JP (2017). Confounding in observational studies based on large health care databases: problems and potential solutions - a primer for the clinician. Clin Epidemiol.

[60] Almasi-Hashiani A, Nedjat S, Ghiasvand R, Safiri S, Nazemipour M, Mansournia N (2021). The causal effect and impact of reproductive factors on breast cancer using super learner and targeted maximum likelihood estimation: a case-control study in Fars province, Iran. BMC Public Health.

[61] Naimi AI, Cole SR, Kennedy EH (2017). An introduction to g methods. Int J Epidemiol.

[62] Keil AP, Edwards JK, Richardson DB, Naimi AI, Cole SR (2014). The parametric g-formula for time-to-event data: intuition and a worked example. Epidemiology.

[63] Taubman SL, Robins JM, Mittleman MA, Hernán MA (2009). Intervening on risk factors for coronary heart disease: an application of the parametric g-formula. Int J Epidemiol.

[64] Jain P, Danaei G, Robins JM, Manson JE, Hernán MA (2016). Smoking cessation and long-term weight gain in the Framingham Heart Study: an application of the parametric g-formula for a continuous outcome. Eur J Epidemiol.

[65] Westreich D, Cole SR, Young JG, Palella F, Tien PC, Kingsley L (2012). The parametric g-formula to estimate the effect of highly active antiretroviral therapy on incident AIDS or death. Stat Med.

[66] Garcia-Aymerich J, Varraso R, Danaei G, Camargo CA Jr, Hernán MA (2014). Incidence of adult-onset asthma after hypothetical interventions on body mass index and physical activity: an application of the parametric g-formula. Am J Epidemiol.

[67] Edwards JK, McGrath LJ, Buckley JP, Schubauer-Berigan MK, Cole SR, Richardson DB (2014). Occupational radon exposure and lung cancer mortality: estimating intervention effects using the parametric g-formula. Epidemiology.

[68] Murray EJ, Robins JM, Seage GR, Freedberg KA, Hernán MA (2017). A comparison of agent-based models and the parametric g-formula for causal inference. Am J Epidemiol.

[69] Young JG, Cain LE, Robins JM, O’Reilly EJ, Hernán MA (2011). Comparative effectiveness of dynamic treatment regimes: an application of the parametric g-formula. Stat Biosci.

[70] Abdollahpour I, Nedjat S, Mansournia MA, Sahraian MA, Kaufman JS (2018). Estimating the marginal causal effect of fish consumption during adolescence on multiple sclerosis: a population-based incident case-control study. Neuroepidemiology.

[71] Mokhayeri Y, Hashemi-Nazari SS, Khodakarim S, Safiri S, Mansournia N, Mansournia MA (2019). Effects of hypothetical interventions on ischemic stroke using parametric g-formula. Stroke.

[72] Mohammadi N, Alimohammadian M, Feizesani A, Poustchi H, Alizadeh A, Yaseri M (2021). The marginal causal effect of opium consumption on the upper gastrointestinal cancer death using parametric g-formula: an analysis of 49,946 cases in the Golestan Cohort Study, Iran. PLoS One.

[73] Mokhayeri Y, Nazemipour M, Mansournia MA, Naimi AI, Kaufman JS (2022). Does weight mediate the effect of smoking on coronary heart disease? Parametric mediational g-formula analysis. PLoS One.

[74] Saatchi M, Mansournia MA, Khalili D, Daroudi R, Yazdani K (2020). Estimation of generalized impact fraction and population attributable fraction of hypertension based on JNC-IV and 2017 ACC/AHA guidelines for cardiovascular diseases using parametric g-formula: Tehran Lipid and Glucose Study (TLGS). Risk Manag Healthc Policy.

[75] Pekmezovic T, Drulovic J, Milenkovic M, Jarebinski M, Stojsavljevic N, Mesaros S (2006). Lifestyle factors and multiple sclerosis: a case-control study in Belgrade. Neuroepidemiology.

[76] Massa J, O’Reilly EJ, Munger KL, Ascherio A (2013). Caffeine and alcohol intakes have no association with risk of multiple sclerosis. Mult Scler.

