# Supplementary material for: Impact of Alcohol Consumption on Multiple Sclerosis Using Model-based Standardization and Misclassification Adjustment Via Probabilistic Bias Analysis
Source: Arch Iran Med. 2023 Oct 1;26(10):567–74. doi: 10.34172/aim.2023.83 (PMC10862089; doi:10.34172/aim.2023.83)
Supplement: Supplementary file 1 — contains Figure S1. [file aim-26-567-s001.pdf]

Arch Iran Med. October 2023;26(10):567-574

10.34172/aim.2023.83

Supplementary file 1

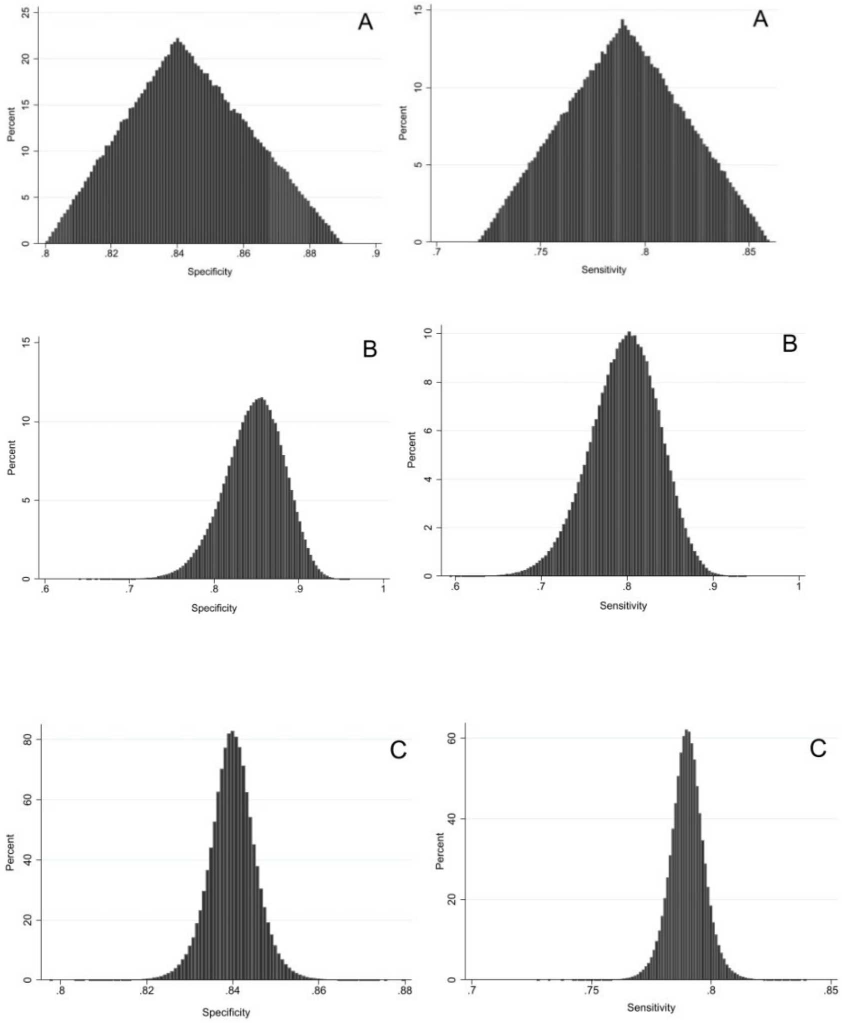

Figure S1. Triangular (A), beta (B) or logit-logistic (C) distributions of specificity and sensitivity.

$$\begin{bmatrix} Sen & 1-Spe \\ 1-Sen & Spe \end{bmatrix} \begin{bmatrix} A \\ B \end{bmatrix} = \begin{bmatrix} A^* \\ B^* \end{bmatrix}$$

The matrix equation presented above illustrates that the observed values in the case group ($\begin{bmatrix} A^* \\ B^* \end{bmatrix}$) are equal to the result of multiplying the sensitivity/specificity matrix ($\begin{bmatrix} Sen & 1 - Spe \\ 1 - Sen & Spe \end{bmatrix}$) by the value of expected exposure in the case group ($\begin{bmatrix} A \\ B \end{bmatrix}$). The equation can be rearranged as follows:

$$\begin{bmatrix} A \\ B \end{bmatrix} = \begin{bmatrix} A^* \\ B^* \end{bmatrix} \begin{bmatrix} Sen & 1 - Spe \\ 1 - Sen & Spe \end{bmatrix}^{-1}$$

Which can be rewritten according to matrix rules:

$$\begin{bmatrix} A \\ B \end{bmatrix} = \begin{bmatrix} \frac{Spe}{Sen + Spe - 1} & \frac{Spe - 1}{Sen + Spe - 1} \\ \frac{Sen - 1}{Sen + Spe - 1} & \frac{Sen}{Sen + Spe - 1} \end{bmatrix} \begin{bmatrix} A^* \\ B^* \end{bmatrix}$$

Therefore, A and B would be calculated using the following formulas:

$$A = \frac{Spe}{Sen + Spe - 1} \times A^* + \frac{Spe - 1}{Sen + Spe - 1} \times B^*$$

$$B = \frac{Sen - 1}{Sen + Spe - 1} \times A^* + \frac{Sen}{Sen + Spe - 1} \times B^*$$

The above calculations should be done for the control group, too.

Then, the positive predictive value (PPV) and negative predictive value (NPV) will be calculated using the following formulas:

$$\text{PPV} = \frac{Sen \times A}{(Sen \times A) + ((1 - Spe) \times B)}$$

$$\text{NPV} = \frac{Spe \times B}{(Spe \times B) + ((1 - Sen) \times A)}$$
